# Elevated Endogenous Erythropoietin Concentrations Are Associated with Increased Risk of Brain Damage in Extremely Preterm Neonates

**DOI:** 10.1371/journal.pone.0115083

**Published:** 2015-03-20

**Authors:** Steven J. Korzeniewski, Elizabeth Allred, J. Wells Logan, Raina N. Fichorova, Stephen Engelke, Karl C. K. Kuban, T. Michael O’Shea, Nigel Paneth, Mari Holm, Olaf Dammann, Alan Leviton, ELGAN study investigators

**Affiliations:** 1 Perinatology Research Branch, NICHD/NIH/DHHS, Bethesda, Maryland, and Detroit, MI, United States of America; 2 Department of Obstetrics and Gynecology, Wayne State University School of Medicine, Detroit, MI, United States of America; 3 Department of Epidemiology & Biostatistics, Michigan State University, East Lansing, MI, United States of America; 4 Neurology Departments, Boston Children’s Hospital, and Harvard Medical School, Boston, MA, United States of America; 5 Department of Neonatology, Nationwide Children’s Hospital, Columbus, OH, United States of America; 6 Departments of Obstetrics, Gynecology and Reproductive Biology, Brigham and Women’s Hospital and Harvard Medical School, Boston, MA, United States of America; 7 Department of Pediatrics, East Carolina University Brody School of Medicine, Greenville, NC, United States of America; 8 Departments of Pediatrics, Boston Medical Center and Boston University, Boston, MA, United States of America; 9 Department of Pediatrics, Wake Forest University, Winston-Salem, NC, United States of America; 10 Department of Pediatrics & Human Development, Michigan State University, East Lansing, MI, United States of America; 11 Department of Laboratory Medicine, Children's and Women's Health, Faculty of Medicine, Norwegian University of Science and Technology, Trondheim, Norway; 12 Department of Public Health and Community Medicine, Tufts University School of Medicine, Boston, MA, United States of America; 13 Neuroepidemiology Unit, Hannover School of Medicine, Hannover, Germany; Robert Debre Hospital, FRANCE

## Abstract

**Background:**

We sought to determine, in very preterm infants, whether elevated perinatal erythropoietin (EPO) concentrations are associated with increased risks of indicators of brain damage, and whether this risk differs by the co-occurrence or absence of intermittent or sustained systemic inflammation (ISSI).

**Methods:**

Protein concentrations were measured in blood collected from 786 infants born before the 28th week of gestation. EPO was measured on postnatal day 14, and 25 inflammation-related proteins were measured weekly during the first 2 postnatal weeks. We defined ISSI as a concentration in the top quartile of each of 25 inflammation-related proteins on two separate days a week apart. Hypererythropoietinemia (hyperEPO) was defined as the highest quartile for gestational age on postnatal day 14. Using logistic regression and multinomial logistic regression models, we compared risks of brain damage among neonates with hyperEPO only, ISSI only, and hyperEPO+ISSI, to those who had neither hyperEPO nor ISSI, adjusting for gestational age.

**Results:**

Newborns with hyperEPO, regardless of ISSI, were more than twice as likely as those without to have very low (< 55) Mental (OR 2.3; 95% CI 1.5-3.5) and/or Psychomotor (OR 2.4; 95% CI 1.6-3.7) Development Indices (MDI, PDI), and microcephaly at age two years (OR 2.4; 95%CI 1.5-3.8). Newborns with both hyperEPO and ISSI had significantly increased risks of ventriculomegaly, hemiparetic cerebral palsy, microcephaly, and MDI and PDI < 55 (ORs ranged from 2.2-6.3), but not hypoechoic lesions or other forms of cerebral palsy, relative to newborns with neither hyperEPO nor ISSI.

**Conclusion:**

hyperEPO, regardless of ISSI, is associated with elevated risks of very low MDI and PDI, and microcephaly, but not with any form of cerebral palsy. Children with both hyperEPO and ISSI are at higher risk than others of very low MDI and PDI, ventriculomegaly, hemiparetic cerebral palsy, and microcephaly.

## Introduction

Just two decades ago, erythropoietin (EPO) receptors were first identified in the brain,[1] and astrocytes were found to be capable of synthesizing EPO.[2] Subsequently, it was found that cultured hippocampal and cerebral cortical neurons exposed to EPO were spared some of the glutamate-induced cell death seen in neurons not exposed to EPO.[3] Thus began the concept that EPO protects the brain against adversity.

Several follow-up studies of children who had participated in trials of recombinant EPO for the prevention or treatment of anemia,[4–6] term newborn encephalopathy,[7] or retinopathy of prematurity[8] have also provided evidence of neuroprotective effects. What has been missing to date, however, is any evidence that *endogenous* levels of EPO in the blood contribute information about the risk of perinatal brain damage.

In the ELGAN (Extremely Low Gestational Age Newborn) study, abnormal brain structure and function were associated with intermittent or sustained systemic inflammation (ISSI).[9–13] Since EPO has anti-inflammatory properties in the kidney [14] and in muscle [15] as well as growth/trophic properties, we reasoned that elevated circulating levels might convey information about reduced risk of brain damage in ELGANs.

In our ELGAN sample, elevated EPO concentrations correlate with higher systemic levels of inflammatory proteins.[16] Consequently, elevated concentrations of endogenous EPO might convey information about inflammation as well as potential neuroprotection. We sought to distinguish between these two possibilities by analyzing associations between elevated endogenous EPO concentrations (defined as concentrations in the highest quartile for gestational age on postnatal day 14) and multiple indicators of brain damage and neurodevelopmental dysfunction, both in the presence and in the absence of ISSI.

## Methods

### The ELGAN Study

The ELGAN study was designed to identify characteristics and exposures that increase the risk of structural and functional neurologic disorders in ELGANs. During the years 2002–2004, women delivering before 28 weeks gestation at one of 14 participating institutions were asked to enroll in the study. Written informed consent was obtained from parents of newborns included in this study using procedures approved by each of the individual hospital institutional review boards listed in the acknowledgement section. Additional details are provided elsewhere[17]. Here, we focus on the contribution of EPO to the occurrence of indicators of perinatal brain damage. Financial limitations allowed us to measure proteins in newborn blood only from the 939 infants who survived and had a developmental evaluation at 24 months adjusted age. The sample presented here consists of the 786 newborns who had protein measurements on protocol day 14.

### Newborn variables

Estimation of gestational age at birth was based on a hierarchy ordered by the quality of available information. Most desirable were estimates based on the dates of embryo retrieval or intrauterine insemination or fetal ultrasound before the 14^th^ week (62%). When these were not available, reliance was placed sequentially on a fetal ultrasound at 14 or more weeks (29%), LMP (7%), and gestational age recorded in the log of the neonatal intensive care unit (1%).

The birth weight Z-score is the number of standard deviations the infant’s birth weight is above or below the median weight of infants at the same gestational age in referent samples not delivered for preeclampsia or fetal indications.[18,19]

Information was collected about blood cultures for each week through the 4^th^ week, but not for each day. Consequently, late bacteremia is defined as being evident during postnatal week 2 (N = 110) and week 3 (N = 75). No child had bacteria first cultured from the blood during week 4. The recovery of an organism from blood was reported, but details about the organism were not. Infection was identified based on documentation of a cultured organism that was considered a potential pathogen and not a contaminant.

### Blood spot collection

Drops of blood were collected on filter paper (Schleicher & Schuell 903) on the first postnatal day (range: 1–3 days), the 7^th^ postnatal day (range: 5–8 days), and the 14^th^ postnatal day (range: 12–15 days), All blood was from the remainder after specimens were obtained for clinical indications. Dried blood spots were stored at -70°C in sealed bags with desiccant until processed.

### Protein measurements

Details about elution of the 25 inflammation-related proteins from blood spots and measurement of the proteins with the Meso Scale Discovery (MSD, Rockville, MD) electrochemiluminescence system are provided elsewhere.[20,21] This electrochemiluminescence system h*as* been validated by comparisons with traditional ELISA, [22,23] and produces measurements that have high content validity, [20,21,24,25] and inter-assay variations that are invariably less than 20%. Because the volume of blood spots can vary in ways that we cannot easily measure, each protein measurement was normalized to mg of total protein. Measurements were made in duplicate and the mean served as the basis for all tables and analyses.

The Laboratory of Genital Tract Biology of the Department of Obstetrics, Gynecology and Reproductive Biology at Brigham and Women's Hospital, Boston, measured the following 25 proteins using commercially available platforms: IL-1beta (Interleukin-1beta), IL-6 (Interleukin-6), IL-6R (interleukin-6 receptor), TNF-alpha (tumor necrosis factor-alpha), TNF-R1 (tumor necrosis factor-alpha-receptor1), TNF-R2 (tumor necrosis factor-alpha-receptor2), IL-8 (CXCL8) (interleukin-8), MCP-1 (CCL2) (monocyte chemotactic protein-1), MCP-4 (CCL13) (monocyte chemoattractant protein-4) (CCL13), MIP-1B (CCL4) (Macrophage Inflammatory Protein-1beta)(CCL4), RANTES (CCL5) (regulated upon activation, normal T-cell expressed, and [presumably] secreted), I-TAC (CXCL11) (Interferon-inducible T cell Alpha-Chemoattractant), ICAM-1 (CD54) (intercellular adhesion molecule-1), ICAM-3 (CD50) (intercellular adhesion molecule-3), VCAM-1 (CD106) (vascular cell adhesion molecule-1), E-SEL (CD62E) (E-selectin) (CD62E), MMP-1 (matrix metalloproteinase-1), MMP-9 (matrix metalloproteinase-9), CRP (C-Reactive Protein), SAA (serum amyloid A), MPO (myeloperoxidase). VEGF (vascular endothelial growth factor), VEGF-R1 (vascular endothelial growth factor-receptor1, Flt-1), VEGF-R2 (vascular endothelial growth factor-receptor2, KDR), and IGFBP-1 (insulin growth factor binding protein-1). Total protein was measured using the BCA assay (Thermo Scientific, Rockford, IL) as previously described.[26]

### Study Groups

Four mutually exclusive study groups were formed based on the presence or absence of relative hypererythropoietinemia (hyperEPO) and intermittent or sustained systemic inflammation (ISSI): 1. hyperEPO only, 2. ISSI only, 3. hyperEPO+ISSI, and 4. neither hyperEPO nor ISSI (referent group).

Newborns with hyperEPO had EPO concentrations in the highest quartile for gestational age on postnatal day 14 (> 25 pg/mg protein among infants born at 23–24 weeks, and > 33–34 pg/mg for those born at 25–27 weeks).

ISSI was defined as a concentration of a specific inflammation-related protein in the top quartile for gestational age, on two separate days, a week apart during the first two postnatal weeks.

### Protocol ultrasound scans

Protocol scans were performed by sonographers at each hospital using high-frequency transducers (7.5 and 10 MHz) and were collected before the 28^th^ postmenstrual week. The cerebral white matter in each hemisphere was divided into eight zones. In each zone, lesions could be further characterized as hyperechoic and/or hypoechoic. Hypoechoic lesion refers to a reduction in echoes measured by ultrasonography that is typically attributed to a presumed pathologic change in tissue density. Ventriculomegaly was defined visually with a template that was on the data collection form. Moderate/severe ventriculomegaly was diagnosed if the lateral ventricle was at least moderately enlarged in any of four sections (frontal horn, body, and occipital horn) on either side. Two independent readers had to agree on the presence of every lesion reported here. Additional details about obtaining and reading ultrasound scans are described elsewhere. [27]

### 24-month developmental assessment

Fully, 91% of surviving children returned for a developmental assessment close to the time when they were 24-months corrected age. The Bayley Scales of Infant Development—Second Edition (BSID-II)[28] were administered and the Mental Development Index (MDI) and the Psychomotor Development Index (PDI) were scored by certified examiners who demonstrated acceptably low variability.[29] Of these children, 77% had their exam within the range of 23.5–27.9 months. Neurological examination data were used for the topographic diagnosis of cerebral palsy (CP) (quadriparesis, diparesis, or hemiparesis) based on an established algorithm.[27] Because newborns were assessed at different approximations of 24 months corrected age, all head circumferences were converted to Z-scores based on standards provided by the CDC.[30] The largest occipital-frontal circumference was measured to the nearest 0.1 centimeter.

### Data analysis

Logistic regression models adjusted for gestational age category (*i*.*e*., 23–24, 25–26, and 27 weeks) were fit to estimate magnitudes of association (odds ratios (OR) with 95% confidence intervals (CI)) between hyperEPO and each of three indicators of brain damage, ultrasound scan diagnoses of ventriculomegaly and a hypoechoic lesion, and at age 2 years, microcephaly. Multinomial logistic regression models that adjusted for gestational age category were fit to estimate associations between hyperEPO and cerebral palsy subtype (quadriparetic, diparetic and hemiparetic, compared to no cerebral palsy) and very low (< 55) and low (55–69) MDI and PDI scores compared to scores of 70 or higher.

Twenty-five additional logistic regression models (one for each inflammation-associated protein) were fit for each of the eight brain damage indicators to evaluate risks associated with hyperEPO only, ISSI only, and the combination of hyperEPO+ISSI. These exposures were compared *in each model* to the absence of both hyperEPO and ISSI. We adjusted for gestational age category and modest fetal growth restriction (birth weight Z-score <-1), finding that the point estimates for the odds ratios differed minimally from those obtained when adjustment was made only for gestational age category. Consequently, we present the odds ratios for gestational age adjustment only.

Risks of cerebral palsy subtypes and very low and low MDI and PDI were evaluated in the same way except the models were, as before, multinomial. Odds ratios and confidence intervals for low (55–69) mental and psychomotor development index scores are shown only in the supplemental tables (Tables F2 & G2 in S1 File).

Odds ratios whose 95% confidence intervals exclude 1.0 are statistically significant.

## Results

### Risks of brain disorders associated with hyperEPO when not considering ISIS (Table 1)

Children with hyperEPO were more than twice as likely as those with lower EPO concentrations to have very low (< 55) MDIs and/or PDIs, or microcephaly at age two years (Table 1). In contrast, newborns with hyperEPO did not have higher frequencies of cranial ultrasound lesions or cerebral palsy than those without.

**Table 1:** Percent of children classified by their EPO concentration for gestational age on postnatal day 14 who also had the characteristics and outcomes listed on the left.

Characteristics	EPO Quartile*			Odds Ratios^†^ for lesion on left associated with hyperEPO	Row N
	Lowest	Middle Two	Highest		
**Ultrasound lesion**					
Ventriculomegaly	9	10	11	1·2 (0·7, 2·0)	79
Hypoechoic lesion	10	7	6	0·8 0·4, 1·5)	58
**Cerebral palsy type^**					
Quadriparesis	6	6	6	0·9 (0·4, 1·8)	48
Diparesis	4	4	3	0·7 (0·3, 1·8)	30
Hemiparesis	3	1	3	2·0 (0·7, 5·6)	15
**Bayley Scales of Infant Development^**					
MDI < 55	8	15	24	**2·3 (1·5, 3·5)**	120
MDI 55–69	9	12	12	1·3 (0·8, 2·2)	88
PDI < 55	11	13	24	**2·4 (1·6, 3·7)**	120
PDI 55–69	10	16	17	1·5 (0·9, 2·3)	116
**Microcephaly**					
HC Z-score < -2	8	8	18	**2·4 (1·5, 3·8)**	84
Column N	198	390	198		786

The odds ratios are for the occurrence of the disorder listed on the left comparing those who had hyperEPO to those who did not.

* These are column percentages, EPO quartiles were determined on day 14

^†^ Odds Ratios (95% confidence intervals) are adjusted for gestational age category

^ cerebral palsy subtype and MDI and PDI category odds ratios were modeled using multinomial logistic regression

### Risks of brain disorders associated with hyperEPO depending the presence or absence of ISIS (Figs. 1–4; Tables A-H in S1 File)

**Fig 1:**
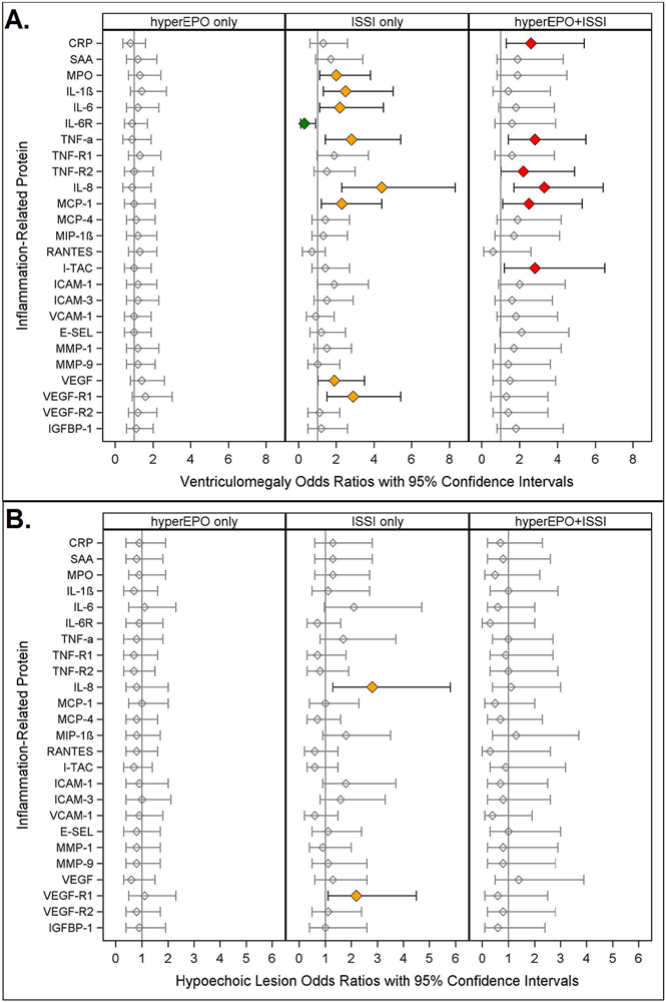
Odds ratios (and 95% confidence intervals) for ventriculomegaly (A.) and hypoechoic lesion (B.) calculated with logistic regression models. The three risk groups: **ISSI only**: (an inflammation-related protein concentration in the highest quartile on two days); **hyperEPO only** (an EPO concentration in the highest quartile on day 14); and **ISSI+hyperEPO** are each compared to the referent group that consists of newborns who had neither ISSI nor hyperEPO. All models are adjusted for gestational age.

**Fig 2:**
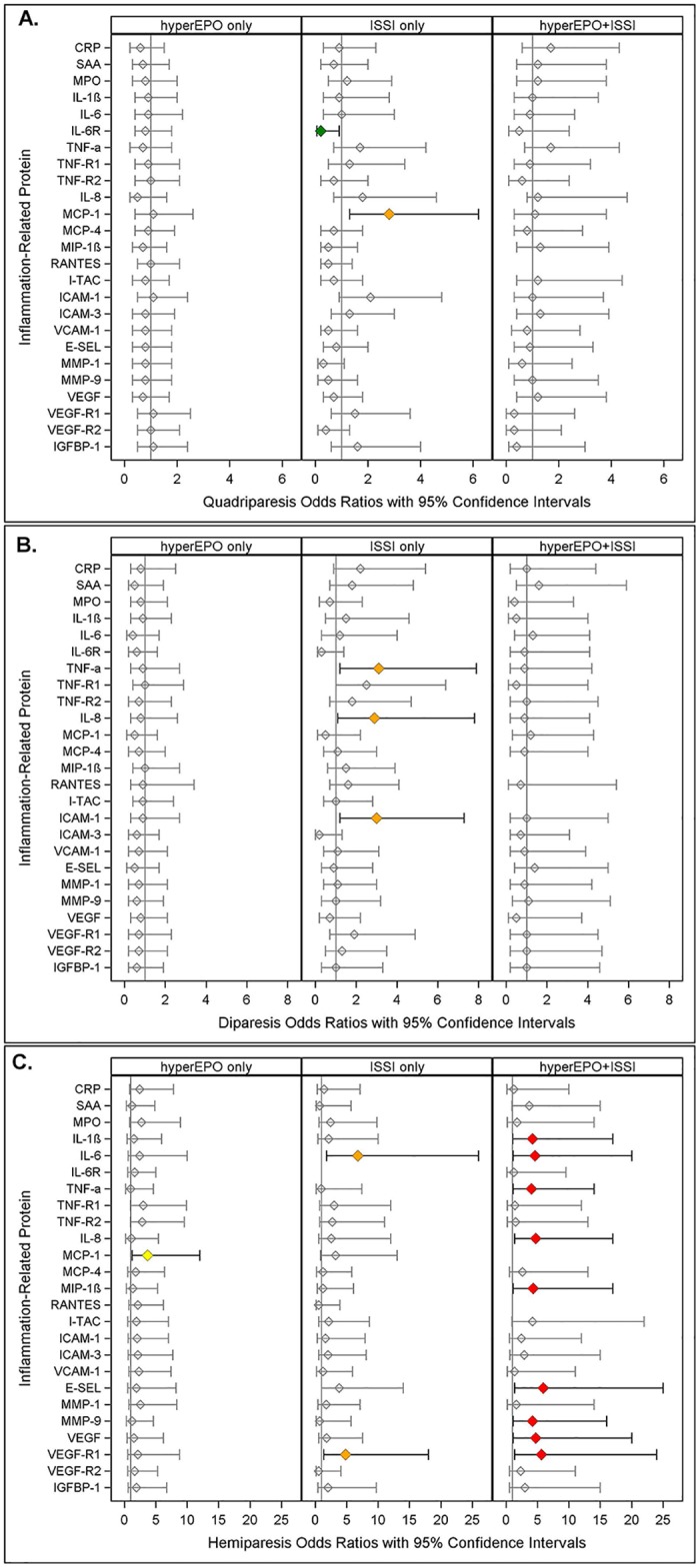
Odds ratios (and 95% confidence intervals) of quadriparesis (A.), diparesis (B.) and hemiparesis (C.) calculated with multinomial logistic regression models with risk groups and adjustment for gestational age as described in Fig. 1. Missing values indicate an inability to estimate odds due to complete separation of outcomes among exposed and unexposed in light of the small sample size.

**Fig 3:**
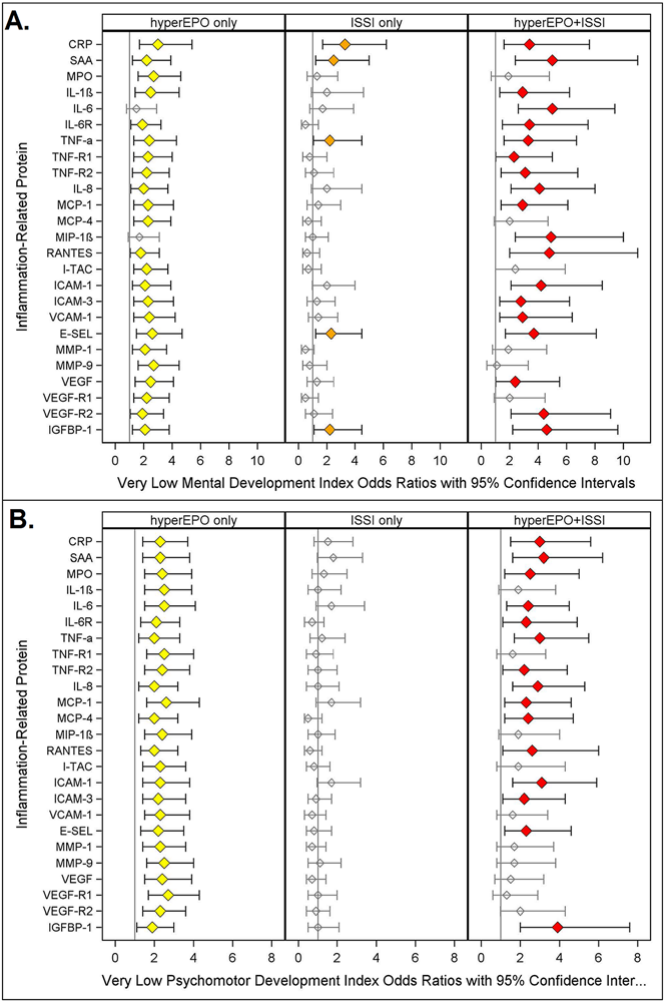
Odds ratios (and 95% confidence intervals) of Very Low Mental Development Index (A.) and Very Low Psychomotor Development Index (B.) calculated with multinomial logistic regression models as described in Fig. 2. Note: Only children with a GMFCS < 1 are included in the MDI analysis.

**Fig 4:**
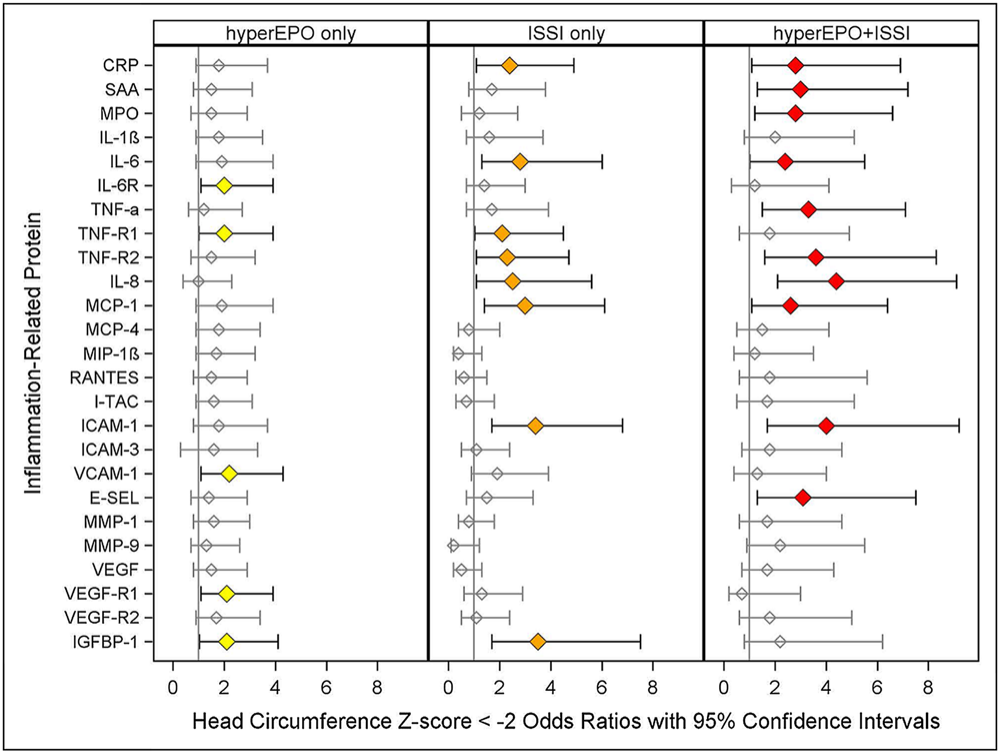
Odds ratios (and 95% confidence intervals) of 24 month head circumference Z-score < -2 calculated with logistic regression models as described in Fig. 1. Note: Children with a birth head circumference Z-score < -2 are excluded from this analysis.

Three patterns of increased risk emerged when we modeled the risk of brain damage associated with hyperEPO depending on the presence or absence of ISSI:

1. hyperEPO only was associated with significantly higher risk,
2. ISSI only was associated with significantly higher (and sometimes lower) risk, and
3. hyperEPO+ISSI was associated with significantly elevated risk.

**Fig 5:**
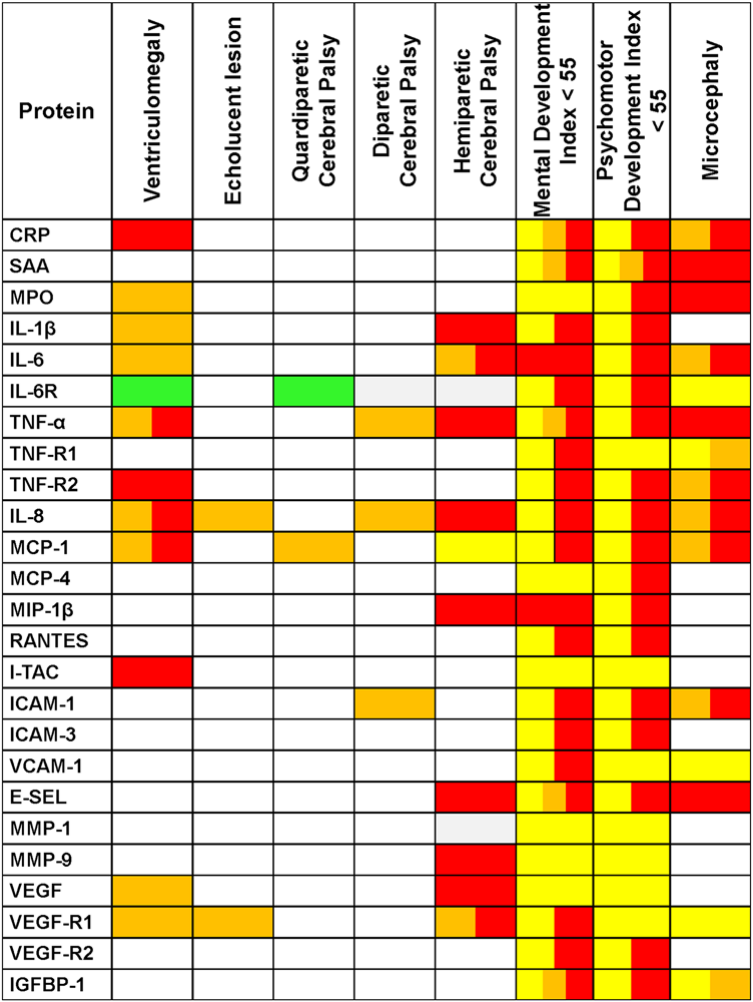
Summary of Associations. We identify three patterns of increased risk of indicators of brain damage associated with hyperEPO and ISSI. hyperEPO only is identified with **yellow**, ISSI **only** is identified with **orange**, and the combination of ISSI+hyperEPO is identified with **red**. Reduced risk of an echolucent lesion associated with hyperEPO **only** is identified with **green**. Boxes with 2 **or 3** separate colors indicate that 2 **or 3** patterns were identified. Note: Cells in this table identify patterns of results of unique multivariable regression models fitted to answer whether or not children in each of three mutually exclusive study groups (hyperEPO only, ISSI only, or hyperEPO+ISSI) were at higher or lower risk of the brain damage indicator identified at the top of each column, relative to those in a referent group who did not have hyperEPO or ISSI, adjusting for gestational age category.

We summarize the application of these three patterns to each protein and indicator of brain damage in a heat map (Fig. 5).

### Ventriculomegaly (Fig. 1A)

Newborns with hyperEPO only were not at significantly different risk of ventriculomegaly, whereas those with ISSI only (MPO, IL-1 β, IL-6, TNF- α, IL-8, MCP-1, VEGF, VEGF-R1, orange) were 2–5 times more likely to have ventriculomegaly than children without either hyperEPO or ISSI.

Newborns with hyperEPO+ISSI (red) were at more than twice the risk of ventriculomegaly as those with neither in six models. For half of these models, neither hyperEPO alone nor ISSI alone was associated with higher risk, yet newborns with hyperEPO+ISSI were at more than twice the risk of ventriculomegaly, when compared to children without either. On the other hand, for five of the models, ISSI only was associated with increased risk, whereas hyperEPO+ISSI was not.

ISSI alone (IL-6R), was associated with significantly *lower* risk of ventriculomegaly.

### Hypoechoic Lesion (Fig. 1B)

In only two of twenty-five models, newborns with ISSI alone (IL-8, VEGF-R1) were at more than twice the risk of hypoechoic lesion when compared to the referent group. Neither hyperEPO alone nor hyperEPO+ISSI was significantly associated with hypoechoic lesions.

### Quadriparetic cerebral palsy (Fig. 2A)

Newborns with hyperEPO alone or hyperEPO+ISSI were not at significantly different risk of quadriparetic cerebral palsy. ISSI alone (MCP-1) was associated with more than twice the risk of quadriparesis, whereas ISSI alone (IL-6R) was associated with significantly *lower* risk, when each was compared to the risk of the referent group.

### Diparetic cerebral palsy (Fig. 2B)

Children with hyperEPO alone or hyperEPO+ISSI were not at significantly elevated risk of diparetic cerebral palsy when compared to referent newborns. ISSI alone (orange), however, was associated with threefold greater risk of diparesis in models for three proteins (TNF-α, IL-8, ICAM-1) when compared to the referent group.

### Hemiparetic cerebral palsy (Fig. 2C)

hyperEPO alone (MCP-1, yellow) was associated with significantly greater risk of hemiparesis, whereas ISSI alone (IL-6 and VEGF-R1, orange) was associated with significantly higher risk compared to referent newborns.

On the other hand, in nine of twenty-five models, hyperEPO+ISSI (ISSI defined by IL-1β, IL-6,TNF-R1,IL-8, MIP-1β, E-SEL, MMP-9, VEGF, VEGF-R1) was associated with more than four-fold greater risk of hemiparetic cerebral palsy (red) compared to the referent group.

### Very low Mental Development Index (MDI)(Fig. 3A)

In all but two of the twenty-five models, newborns with hyperEPO alone (yellow) had a two- to three-fold greater risk of MDI < 55 than those in the referent group. In nineteen of the twenty-five models, hyperEPO+ISSI (red) was associated with a two to six times greater risk of MDI < 55 than newborns in the referent group.

In five models, newborns with ISSI alone (CRP, SAA, TNF-α, E-SEL, IGFBP-1, orange) were at two to three times greater risk of MDI < 55 when compared to referent newborns.

Neither hyperEPO alone nor ISSI alone was associated with increased risk of low MDI (55–69), whereas hyperEPO+ISSI was associated with a two- to three-fold increased risk in four of twenty-five models, when newborns with each condition were compared to referent children (Table F2 in S1 File).

### Very low Psychomotor Development Index (PDI)(Fig. 3B)

In all twenty-five models, newborns with hyperEPO alone (yellow) were at more than twice the risk of PDI < 55 compared to referent children. In 18 models, hyperEPO+ISSI (red) was associated with a two- to four-fold greater risk of a PDI < 55, whereas newborns with ISSI alone were at a twofold greater risk of PDI < 55 in one model (SAA).

HyperEPO+ISSI was associated with a two- to three-fold greater risk of PDI between 55 and 69 in fifteen of the twenty-five models, when newborns with each condition were compared to referent children, while there were essentially no associations with hyperEPO alone or ISSI alone (Table G2 in S1 File).

### Microcephaly (Fig. 4)

In three models, newborns with hyperEPO alone (IL-6R, VCAM-1, VEGF-R1, yellow) were at significantly greater risk of microcephaly than referent children. In eight models, ISSI alone (CRP, IL-6, TNF-R1, TNF-R2, IL-8, MCP-1, ICAM-1, IGFBP-1, orange) was also associated with a two to four fold greater risk of microcephaly, compared to referent newborns. The risk of microcephaly was increased in newborns with hyperEPO+ISSI (ISSI defined by CRP, SAA, MPO, IL-6, TNF-α, TNF-R2, IL-8, MCP-1, ICAM-1, E-SEL, red), often more prominently than for ISSI alone.

## Discussion

Four of our findings are worthy of comment.

1. HyperEPO, when considered alone, conveyed information about significantly elevated risks of neurodevelopmental dysfunction (MDI and PDI < 55) and microcephaly.
2. The risks of these outcomes were often even higher when hyperEPO was considered in combination with ISSI.
3. When evaluated in combination with ISSI, hyperEPO was also associated with significantly elevated risks of ventriculomegaly and hemiparesis, but not other forms, of cerebral palsy.
4. ISSI, when considered in light of information about hyperEPO, was associated with ventriculomegaly and microcephaly more so than with very low MDI.

Our first finding ignores ISSI entirely. Simply, hyperEPO, regardless of inflammatory state, conveyed information about three entities that had previously been associated with ISSI, very low MDI and PDI, and microcephaly.

When we considered, simultaneously, combinations of hyperEPO and ISSI (hyperEPO only, ISSI only, hyperEPO+ISSI, no hyperEPO or ISSI), we found that hyperEPO only was fairly consistently associated with increased risk of very low MDI and PDI, and that hyperEPO+ISSI was associated with considerably higher risks of these developmental dysfunctions. In short, hyperEPO+ISSI appears more strongly associated with brain damage and neurodevelopmental dysfunction than hyperEPO alone or ISSI alone.

The association of a sustained increase of IL-6R with lower frequencies of ventriculomegaly and quadriparesis might constitute a chance finding since the same was not observed for any of the other 24 inflammation-related proteins in this study. However, it is possible that IL-6R might protect against some forms of brain damage since the classic IL-6 signaling via its membrane receptor IL-6R can have anti-inflammatory and tissue regenerating effects [31] and we had previously found a lower likelihood of motor impairment [32] and quadriparesis [33] in ELGANS with elevated IL-6R.

Our findings of increased risk of functional and structural indicators of brain damage associated with hyperEPO do not preclude the possibility that EPO treatment might prevent adverse neurodevelopmental outcomes, as has been suggested in some observational studies,[5,6] but indicates a need for caution in the development of trials testing effects of exogenously administered EPO, which is likely to result in considerably higher blood, and perhaps brain concentrations.[34] In addition, the relationship between endogenous EPO concentrations in blood and brain remains unclear.

### Synthesis

It is unclear how hyperEPO conveys information about or actually contributes to brain damage. EPO may influence brain damage risk through a number of its pleiotropic effects, including stimulation of neuro- and angio-genesis,[35–44] or anti-inflammatory actions.[45–49] hyperEPO might also contribute to brain damage via inflammatory phenomena.[16] Another possibility is that hyperEPO adds information about maturity/vulnerability beyond that provided by gestational age at delivery, thus reflecting immaturity either of the brain itself, or of the systems that have the capacity to protect it. Alternatively, unmeasured factors, for instance the severity of the insult prompting the initial inflammatory response, may be correlated with EPO and lead to a non-causal association between hyperEPO and brain damage. Indeed, we view each of these possibilities as plausible.

Unfortunately, we did not record hemoglobin or hematocrit levels. Consequently, we cannot evaluate if the elevated EPO concentrations are a consequence of anemia. Most of the babies in our study received weekly transfusions. Using the receipt of a transfusion as an indicator of the need for a transfusion, we did not find that those with the highest concentrations of EPO were more likely than others to receive a transfusion during the first two postnatal weeks (88% vs 86%). We feel reasonably comfortable that anemia is not the driving force behind elevated EPO concentrations in the blood. More likely is the possibility that inflammatory phenomena contribute appreciably to the occurrence of hyperEPO.[16]

### Strengths and limitations

The strengths of our study are: i) the large number of infants providing power to perceive a doubling or halving of risk, ii) enrollment of infants based on gestational age and not birth weight [50], iii) the efforts to reduce observer variability; and iv) the high quality of the protein data.[20–25]

Limitations deserving attention include the following. First, children who died with brain damage prior to developmental assessments at age 2 years are not included; if they differ from survivors, bias may have been introduced. Second, as with all observational studies, we are limited in our ability to infer causation from associations. Third, the proteins we measured probably represent only a fraction of those involved in the genesis of perinatal brain damage. Fourth, in our desire to avoid the error of inappropriately drawing the inference that hyperEPO has no effect, we did not adjust for multiple comparisons, possibly increasing the probability of a Type I error. However, with 95% confidence intervals, only 30 of the reported 600 odds ratios (i.e., 5%) are expected to be statistically significant by chance alone. We found that 144 ORs characterized statistically significant associations—about five times more than expected. Thus, our findings are highly unlikely to reflect random phenomena.

## Conclusion

hyperEPO in very preterm newborns is associated with elevated risks of functional and structural indicators of brain damage. The pattern of risks differs, however, when hyperEPO is considered in the absence and, especially, the presence of ISSI. We cannot identify to what extent the hyperEPO reflects inflammation, immaturity of neuroprotective systems, or immaturity/vulnerability of the brain, or other reasons for its association with brain-related outcomes.

## Supporting Information

S1 File Odds ratios (and 95% confidence intervals) of perinatal brain damage indicators calculated with logistic regression models as described in Figs. 1–5. The three risk groups: ISSI only: (an inflammation-related protein concentration in the highest quartile on two days); hyperEPO only (an EPO concentration in the highest quartile on day 14); and ISSI+hyperEPO are each compared to the referent group that consists of newborns who had neither ISSI nor hyperEPO. Risks of cerebral palsy and MDI/PDI subclassificaitons were modeled using multinomial logistic regression. All models are adjusted for gestational age.
(DOC)
