# Supplementary material for: Elevated Endogenous Erythropoietin Concentrations Are Associated with Increased Risk of Brain Damage in Extremely Preterm Neonates
Source: PLoS One. 2015 Mar 20;10(3):e0115083. doi: 10.1371/journal.pone.0115083 (PMC4368546; doi:10.1371/journal.pone.0115083)
Supplement: S1 File — The three risk groups: ISSI only: (an inflammation-related protein concentration in the highest quartile on two days); hyperEPO only (an EPO concentration in the highest quartile on day 14); and ISSI+hyperEPO are each compared to the referent group that consists of newborns who had neither ISSI nor hyperEPO. Risks of cerebral palsy and MDI/PDI subclassificaitons were modeled using multinomial logistic regression. All models are adjusted for gestational age. (DOC) [file pone.0115083.s001.doc]

**Table A**. Odds ratios (and 95% confidence intervals) of ventriculomegaly calculated with logistic regression models. The three risk groups: ISSI only: (an inflammation-related protein concentration in the highest quartile on two days); hyperEPO only (an EPO concentration in the highest quartile on day 14); and ISSI+hyperEPO are each compared to the referent group that consists of newborns who had neither ISSI nor hyperEPO. All models are adjusted for gestational age.

| **Exposure**  **Protein** | **hyperEPO only** | **ISSI only** | **hyperEPO+ISSI** |
| --- | --- | --- | --- |
| CRP | 0.8 (0.4, 1.6) | 1.3 (0.6, 2.6) | **2.6 (1.3, 5.4)** |
| SAA | 1.2 (0.6, 2.2) | 1.7 (0.9, 3.4) | 1.9 (0.8, 4.3) |
| MPO | 1.3 (0.7, 2.4) | **2.0 (1.1, 3.8)** | 1.9 (0.8, 4.5) |
| IL-1β | 1.4 (0.8, 2.7) | **2.5 (1.3, 5.0)** | 1.4 (0.6, 3.6) |
| IL-6 | 1.2 (0.6, 2.3) | **2.2 (1.1, 4.5)** | 1.8 (0.9, 3.8) |
| IL-6R | 0.9 (0.5, 1.7) | ***0.3 (0.1, 0.9)*** | 1.6 (0.7, 3.9) |
| TNF-α | 0.9 (0.4, 1.9) | **2.8 (1.4, 5.4)** | **2.8 (1.4, 5.5)** |
| TNF-R1 | 1.3 (0.7, 2.4) | 1.9 (0.97, 3.7) | 1.6 (0.7, 3.8) |
| TNF-R2 | 1.0 (0.5, 2.0) | 1.5 (0.8, 3.0) | **2.2 (1.01, 4.9)** |
| IL-8 (CXCL8) | 0.9 (0.4, 1.9) | **4.4 (2.3, 8.3)** | **3.3 (1.7, 6.4)** |
| MCP-1 (CCL2) | 1.0 (0.5, 2.1) | **2.3 (1.2, 4.4)** | **2.5 (1.1, 5.3)** |
| MCP-4 (CCL13) | 1.1 (0.6, 2.1) | 1.4 (0.7, 2.7) | 1.9 (0.8, 4.2) |
| MIP-1β *(*CCL4) | 1.2 (0.6, 2.2) | 1.3 (0.7, 2.6) | 1.7 (0.7, 4.1) |
| RANTES (CCL5) | 1.3 (0.7, 2.2) | 0.7 (0.2, 1.4) | 0.6 (0.1, 2.6) |
| I-TAC ( CXCL11) | 1.0 (0.5, 1.9) | 1.4 (0.7, 2.7) | **2.8 (1.2, 6.5)** |
| ICAM-1 (CD54) | 1.2 (0.6, 2.2) | 1.9 (0.99, 3.7) | 2.0 (0.9, 4.4) |
| ICAM-3 (CD50) | 1.2 (0.6, 2.3) | 1.5 (0.8, 2.9) | 1.6 (0.7, 3.7) |
| VCAM-1 (CD106) | 1.0 (0.5, 1.9) | 0.9 (0.4, 1.9) | 1.8 (0.8, 4.0) |
| E-SEL (CD62E) | 1.0 (0.5, 1.9) | 1.2 (0.6, 2.5) | 2.1 (0.96, 4.6) |
| MMP-1 | 1.2 (0.6, 2.3) | 1.5 (0.8, 2.8) | 1.7 (0.7, 4.2) |
| MMP-9 | 1.2 (0.6, 2.1) | 1.0 (0.5, 2.2) | 1.4 (0.6, 3.6) |
| VEGF | 1.4 (0.8, 2.6) | **1.9 (1.01, 3.5)** | 1.5 (0.6, 3.9) |
| VEGF-R1 | 1.6 (0.9, 3.0) | **2.9 (1.5, 5.4)** | 1.3 (0.5, 3.5) |
| VEGF-R2 | 1.2 (0.7, 2.2) | 1.1 (0.5, 2.2) | 1.4 (0.6, 3.5) |
| IGFBP-1 | 1.1 (0.6, 2.0) | 1.2 (0.5, 2.6) | 1.8 (0.8, 4.3) |

**Table B**. Odds ratios (and 95% confidence intervals) of **a hypoechoic lesion** calculated with logistic regression models as described in Figure 1

| **Exposure**  **protein** | **hyperEPO only** | **ISSI only** | **hyperEPO+ISSI** |
| --- | --- | --- | --- |
| CRP | 0.9 (0.4, 1.9) | 1.3 (0.6, 2.8) | 0.7 (0.2, 2.3) |
| SAA | 0.8 (0.4, 1.8) | 1.3 (0.6, 2.8) | 0.8 (0.2,2.6) |
| MPO | 0.9 (0.5, 1.9) | 1.3 (0.6, 2.7) | 0.5 (0.1, 2.2) |
| IL-1β | 0.7 (0.3, 1.6) | 1.1 (0.5, 2.7) | 1.0 (0.3, 2.9) |
| IL-6 | 1.1 (0.5, 2.3) | 2.1 (0.97, 4.7) | 0.6 (0.2, 2.0) |
| IL-6R | 0.9 (0.4, 1.8) | 0.7 (0.3, 1.6) | 0.3 (0, 2.0) |
| TNF-α | 0.8 (0.3, 1.8) | 1.7 (0.8, 3.7) | 1.0 (0.4, 2.7) |
| TNF-R1 | 0.7 (0.3, 1.6) | 0.7 (0.3, 1.8) | 0.9 (0.3, 2.7) |
| TNF-R2 | 0.7 (0.3, 1.5) | 0.8 (0.3, 1.9) | 1.0 (0.3, 2.9) |
| IL-8 (CXCL8) | 0.8 (0.4, 2.0) | **2.8 (1.3, 5.8)** | 1.1 (0.4, 3.0) |
| MCP-1 (CCL2) | 1.0 (0.5, 2.0) | 1.0 (0.4, 2.3) | 0.5 (0.1, 2.0) |
| MCP-4 (CCL13) | 0.8 (0.4, 1.6) | 0.7 (0.3, 1.6) | 0.7 (0.2, 2.3) |
| MIP-1β *(*CCL4) | 0.8 (0.4, 1.7) | 1.8 (0.9, 3.5) | 1.3 (0.4, 3.7) |
| RANTES (CCL5) | 0.8 (0.4, 1.6) | 0.6 (0.2, 1.5) | 0.3 (0, 2.6) |
| I-TAC ( CXCL11) | 0.7 (0.3, 1.4) | 0.6 (0.3, 1.5) | 0.9 (0.3, 3.2) |
| ICAM-1 (CD54) | 0.9 (0.4, 2.0) | 1.8 (0.9, 3.7) | 0.7 (0.2, 2.5) |
| ICAM-3 (CD50) | 1.0 (0.4, 2.1) | 1.6 (0.8, 3.3) | 0.8 (0.2, 2.6) |
| VCAM-1 (CD106) | 0.9 (0.4, 1.8) | 0.6 (0.2, 1.5) | 0.4 (0.1, 1.9) |
| E-SEL (CD62E) | 0.8 (0.3, 1.7) | 1.1 (0.5, 2.4) | 1.0 (0.3, 3.0) |
| MMP-1 | 0.8 (0.4, 1.7) | 0.9 (0.4, 2.0) | 0.8 (0.2, 2.9) |
| MMP-9 | 0.8 (0.4, 1.7) | 1.1 (0.5, 2.6) | 0.8 (0.2, 2.8) |
| VEGF | 0.6 (0.3, 1.5) | 1.3 (0.6, 2.6) | 1.4 (0.5, 3.9) |
| VEGF-R1 | 1.1 (0.5, 2.3) | **2.2 (1.1, 4.5)** | 0.6 (0.1, 2.5) |
| VEGF-R2 | 0.8 (0.4, 1.7) | 1.1 (0.5, 2.4) | 0.8 (0.2, 2.8) |
| IGFBP-1 | 0.9 (0.4, 1.9) | 1.0 (0.4, 2.6) | 0.6 (0.1, 2.4) |

**Table C**. Odds ratios (and 95% confidence intervals) of **quadriparesis** calculated with multinomial logistic regression models as described in Figure 2.

| **Exposure**  **protein** | **hyperEPO only** | **ISSI only** | **hyperEPO+ISSI** |
| --- | --- | --- | --- |
| CRP | 0.6 (0.2, 1.5) | 0.9 (0.3, 2.3) | 1.7 (0.6, 4.3) |
| SAA | 0.7 (0.3, 1.7) | 0.7 (0.2, 2.0) | 1.2 (0.4, 3.8) |
| MPO | 0.8 (0.3, 2.0) | 1.2 (0.5, 2.9) | 1.2 (0.4, 3.8) |
| IL-1β | 0.9 (0.4, 2.0) | 0.9 (0.3, 2.8) | 1.0 (0.3, 3.5) |
| IL-6 | 0.9 (0.4, 2.2) | 1.0 (0.3, 3.0) | 0.9 (0.3, 2.6) |
| IL-6R | 0.8 (0.4, 1.8) | ***0.2 (0.05, 0.9)*** | 0.5 (0.1, 2.4) |
| TNF-α | 0.7 (0.2, 1.8) | 1.7 (0.7, 4.2) | 1.7 (0.7, 4.3) |
| TNF-R1 | 0.9 (0.4, 2.1) | 1.3 (0.5, 3.4) | 0.9 (0.3, 3.2) |
| TNF-R2 | 1.0 (0.4, 2.1) | 0.7 (0.2, 2.0) | 0.6 (0.1, 2.4) |
| IL-8 (CXCL8) | 0.5 (0.2, 1.6) | 1.8 (0.7, 4.6) | 1.2 (0.8, 4.6) |
| MCP-1 (CCL2) | 1.1 (0.4, 2.6) | **2.8 (1.3, 6.2)** | 1.1 (0.3, 3.8) |
| MCP-4 (CCL13) | 0.9 (0.4, 1.9) | 0.7 (0.2, 1.8) | 0.8 (0.3, 2.9) |
| MIP-1β *(*CCL4) | 0.7 (0.3, 1.6) | 0.5 (0.2, 1.6) | 1.3 (0.4, 3.9) |
| RANTES (CCL5) | 1.0 (0.5, 2.1) | 0.5 (0.2, 1.4) | ---- |
| I-TAC ( CXCL11) | 0.8 (0.3, 1.7) | 0.7 (0.2, 1.8) | 1.2 (0.4, 4.4) |
| ICAM-1 (CD54) | 1.1 (0.5, 2.4) | 2.1 (0.9, 4.8) | 1.0 (0.3, 3.7) |
| ICAM-3 (CD50) | 0.8 (0.3, 1.9) | 1.3 (0.6, 3.0) | 1.3 (0.4, 3.9) |
| VCAM-1 (CD106) | 0.8 (0.3, 1.8) | 0.5 (0.2, 1.6) | 0.8 (0.2, 2.8) |
| E-SEL (CD62E) | 0.8 (0.3, 1.8) | 0.8 (0.3, 2.0) | 0.9 (0.3, 3.3) |
| MMP-1 | 0.8 (0.3, 1.8) | 0.3 (0.1, 1.1) | 0.6 (0.1, 2.5) |
| MMP-9 | 0.8 (0.3, 1.8) | 0.5 (0.1, 1.6) | 1.0 (0.3, 3.5) |
| VEGF | 0.7 (0.3, 1.7) | 0.7 (0.3, 1.8) | 1.2 (0.4, 3.8) |
| VEGF-R1 | 1.1 (0.5, 2.5) | 1.5 (0.6, 3.6) | 0.3 (0, 2.6) |
| VEGF-R2 | 1.0 (0.5, 2.1) | 0.4 (0.1, 1.3) | 0.3 (0, 2.1) |
| IGFBP-1 | 1.1 (0.5, 2.4) | 1.6 (0.6, 4.0) | 0.4 (0.1, 3.) |

**Table D.** Odds ratios (and 95% confidence intervals) of **diparesis** calculated with multinomial logistic regression models as described in Figure 2.

| **Exposure**  **protein** | **hyperEPO only** | **ISSI only** | **hyperEPO+ISSI** |
| --- | --- | --- | --- |
| CRP | 0.8 (0.3, 2.5) | 2.2 (0.9, 5.4) | 1.0 (0.2, 4.4) |
| SAA | 0.5 (0.2, 1.9) | 1.8 (0.7, 4.8) | 1.6 (0.5, 5.9) |
| MPO | 0.8 (0.3, 2.1) | 0.7 (0.2, 2.3) | 0.4 (0.1, 3.3) |
| IL-1β | 0.9 (0.3, 2.3) | 1.5 (0.5, 4.6) | 0.5 (0.1, 4.0) |
| IL-6 | 0.4 (0.1, 1.7) | 1.2 (0.3, 4.0) | 1.3 (0.4, 4.1) |
| IL-6R | 0.6 (0.2, 1.6) | 0.3 (0.1, 1.4) | 0.9 (0.2, 4.1) |
| TNF-α | 0.9 (0.3, 2.7) | **3.1 (1.2, 7.9)** | 0.9 (0.2, 4.2) |
| TNF-R1 | 1.0 (0.4, 2.9) | 2.5 (1.00, 6.4) | 0.5 (0.1, 4.0) |
| TNF-R2 | 0.7 (0.2, 2.3) | 1.8 (0.7, 4.7) | 1.0 (0.2, 4.5) |
| IL-8 (CXCL8) | 0.8 (0.3, 2.6) | **2.9 (1.1, 7.8)** | 0.9 (0.2, 4.1) |
| MCP-1 (CCL2) | 0.5 (0.1, 1.6) | 0.5 (0.1, 2.2) | 1.2 (0.3, 4.3) |
| MCP-4 (CCL13) | 0.7 (0.2, 2.0) | 1.1 (0.4, 3.0) | 0.9 (0.2, 4.0) |
| MIP-1β *(*CCL4) | 1.0 (0.4, 2.7) | 1.5 (0.6, 3.9) | ---- |
| RANTES (CCL5) | 0.9 (0.3, 3.4) | 1.6 (0.7, 4.1) | 0.7 (0.1, 5.4) |
| I-TAC ( CXCL11) | 0.9 (0.4, 2.4) | 1.0 (0.4, 2.8) | ---- |
| ICAM-1 (CD54) | 0.9 (0.3, 2.7) | **3.0 (1.2, 7.3)** | 1.0 (0.2, 5.0) |
| ICAM-3 (CD50) | 0.6 (0.2, 1.7) | 0.2 (0, 1.3) | 0.7 (0.2, 3.1) |
| VCAM-1 (CD106) | 0.7 (0.2, 2.1) | 1.1 (0.4, 3.1) | 0.9 (0.2, 3.9) |
| E-SEL (CD62E) | 0.5 (0.1, 1.7) | 0.9 (0.3, 2.8) | 1.4 (0.4, 5.0) |
| MMP-1 | 0.7 (0.2, 2.1) | 1.1 (0.4, 3.0) | 0.9 (0.2, 4.2) |
| MMP-9 | 0.6 (0.2, 1.9) | 1.0 (0.3, 3.2) | 1.1 (0.3, 5.1) |
| VEGF | 0.8 (0.3, 2.1) | 0.7 (0.2, 2.2) | 0.5 (0.1, 3.7) |
| VEGF-R1 | 0.7 (0.2, 2.3) | 1.9 (0.7, 4.9) | 1.0 (0.2, 4.5) |
| VEGF-R2 | 0.7 (0.2, 2.1) | 1.3 (0.5, 3.5) | 1.0 (0.2, 4.7) |
| IGFBP-1 | 0.6 (0.2, 1.9) | 1.0 (0.3, 3.3) | 1.0 (0.2, 4.6) |

**Table E**. Odds ratios (and 95% confidence intervals) of **hemiparesis** calculated with multinomial logistic regression models as described in Figure 2.

| **Exposure**  **protein** | **hyperEPO only** | **ISSI only** | **hyperEPO+ISSI** |
| --- | --- | --- | --- |
| CRP | 2.4 (0.8, 7.8) | 1.4 (0.3, 7.1) | 1.2 (0.1, 10) |
| SAA | 1.2 (0.3, 4.8) | 0.7 (0.1, 5.7) | 3.7 (0.95, 15) |
| MPO | 2.7 (0.8, 8.9) | 2.4 (0.6, 9.8) | 1.7 (0.2, 14) |
| IL-1β | 1.5 (0.4, 5.9) | 2.1 (0.4, 10) | **4.2 (1.04, 17)** |
| IL-6 | 2.4 (0.6, 10) | **6.8 (1.8, 26)** | **4.6 (1.1, 20)** |
| IL-6R | 1.6 (0.5, 5.0) | ---- | 1.2 (0.1, 9.5) |
| TNF-α | 1.0 (0.2, 4.6) | 0.9 (0.1, 7.4) | **4.0 (1.1, 14)** |
| TNF-R1 | 3.0 (0.9, 9.9) | 3.0 (0.7, 12) | 1.4 (0.2, 12) |
| TNF-R2 | 2.8 (0.9, 9.5) | 2.7 (0.7, 11) | 1.5 (0.2, 13) |
| IL-8 (CXCL8) | 1.1 (0.2, 5.4) | 2.5 (0.5, 12) | **4.7 (1.3, 17)** |
| MCP-1 (CCL2) | **3.7 (1.2, 12)** | 3.2 (0.8, 13) | ---- |
| MCP-4 (CCL13) | 1.8 (0.5, 6.4) | 1.2 (0.2, 5.8) | 2.6 (0.5, 13) |
| MIP-1β *(*CCL4) | 1.3 (0.3, 5.3) | 1.2 (0.3, 6.1) | **4.3 (1.1, 17)** |
| RANTES (CCL5) | 2.1 (0.7, 6.2) | 0.5 (0.1, 3.9) | ---- |
| I-TAC ( CXCL11) | 1.9 (0.5, 7.0) | 2.1 (0.5, 8.6) | 4.2 (0.9, 22) |
| ICAM-1 (CD54) | 2.0 (0.6, 7.0) | 1.6 (0.3, 7.9) | 2.4 (0.5, 12) |
| ICAM-3 (CD50) | 2.1 (0.6, 7.7) | 2.0 (0.5, 8.1) | 2.9 (0.6, 15) |
| VCAM-1 (CD106) | 2.3 (0.7, 7.4) | 1.2 (0.2, 5.9) | 1.3 (0.2, 11) |
| E-SEL (CD62E) | 1.9 (0.5, 8.2) | 3.8 (0.99, 14) | **5.9 (1.3, 25)** |
| MMP-1 | 2.5 (0.7, 8.3) | 1.7 (0.4, 7.1) | 1.6 (0.2, 14) |
| MMP-9 | 1.2 (0.3, 4.6) | 0.7 (0.1, 5.7) | **4.2 (1.1, 16)** |
| VEGF | 1.5 (0.4, 6.2) | 1.8 (0.5, 7.5) | **4.7 (1.1, 20)** |
| VEGF-R1 | 2.1 (0.5, 8.8) | **4.8 (1.3, 18)** | **5.6 (1.3, 24)** |
| VEGF-R2 | 1.6 (0.5, 5.3) | 0.5 (0.1, 4.1) | 2.3 (0.5, 11) |
| IGFBP-1 | 1.9 (0.6, 6.7) | 2.0 (0.4, 9.7) | 3.0 (0.5, 15) |

**Table F1. Odds ratios (and 95% confidence intervals) of MDI < 55 (when GMFCS < 1) calculated with multinomial logistic regression models as described in Figure 2.**

| **Exposure**  **protein** | **hyperEPO only** | **ISSI only** | **hyperEPO+ISSI** |
| --- | --- | --- | --- |
| CRP | **3.1 (1.7, 5.6)** | **3.5 (1.8, 6.7)** | **3.7 (1.7, 8.3)** |
| SAA | **2.2 (1.2, 4.0)** | **2.6 (1.3, 5.3)** | **6.3 (2.9, 14)** |
| MPO | **2.8 (1.6, 4.8)** | 1.3 (0.6, 2.7) | 1.9 (0.7, 4.9) |
| IL-1β | **2.6 (1.5, 4.6)** | 1.9 (0.9, 4.0) | **3.0 (1.4, 6.7)** |
| IL-6 | 1.6 (0.8, 3.0) | 1.7 (0.7, 3.8) | **5.3 (2.7, 10)** |
| IL-6R | **1.9 (1.1, 3.4)** | 0.6 (0.2, 1.4) | **3.4 (1.5, 7.5)** |
| TNF-α | **2.4 (1.3, 4.4)** | **2.2 (1.1, 4.7)** | **3.6 (1.8, 7.5)** |
| TNF-R1 | **2.3 (1.3, 4.1)** | 0.8 (0.3, 1.9) | **2.4 (1.1, 5.4)** |
| TNF-R2 | **2.2 (1.3, 3.9)** | 1.2 (0.5, 2.7) | **3.7 (1.6, 8.2)** |
| IL-8 (CXCL8) | **2.0 (1.1, 3.7)** | 2.0 (0.9 , 4.5) | **4.7 (1.2, 9.5)** |
| MCP-1 (CCL2) | **2.4 (1.4, 4.3)** | 1.4 (0.6, 3.0) | **2.9 (1.4, 6.3)** |
| MCP-4 (CCL13) | **2.4 (1.4, 4.1)** | 0.7 (0.3, 1.5) | 2.0 (0.9, 4.6) |
| MIP-1β *(*CCL4) | 1.7 (0.96, 3.2) | 1.0 (0.5, 2.2) | **5.4 (2.6, 11)** |
| RANTES (CCL5) | **1.8 (1.1, 3.2)** | 0.6 (0.3, 1.3) | **4.9 (2.0, 12)** |
| I-TAC ( CXCL11) | **2.3 (1.3, 3.9)** | 0.7 (0.3, 1.6) | 2.3 (0.9, 5.6) |
| ICAM-1 (CD54) | **2.2 (1.2, 4.0)** | 2.0 (0.98, 4.1) | **4.5 (2.2, 9.3)** |
| ICAM-3 (CD50) | **2.4 (1.3, 4.2)** | 1.2 (0.6, 2.4) | **3.0 (1.4, 6.6)** |
| VCAM-1 (CD106) | **2.5 (1.4, 4.5)** | 1.4 (0.7, 2.9) | **2.8 (1.3, 6.2)** |
| E-SEL (CD62E) | **2.6 (1.5, 4.6)** | **2.4 (1.2, 4.7)** | **4.5 (2.0, 10)** |
| MMP-1 | **2.2 (1.3, 3.8)** | 0.5 (0.1, 1.1) | 1.8 (0.7, 4.4) |
| MMP-9 | **2.8 (1.7, 4.8)** | 0.8 (0.3, 2.0) | 1.1 (0.4, 3.2) |
| VEGF | **2.6 (1.5, 4.6)** | 1.1 (0.6, 2.3) | 2.3 (0.96, 5.3) |
| VEGF-R1 | **2.2 (1.3, 3.8)** | 0.5 (0.2, 1.3) | **2.3 (1.02, 5.1)** |
| VEGF-R2 | **1.9 (1.1, 3.5)** | 1.1 (0.4, 2.4) | **4.6 (2.2, 9.8)** |
| IGFBP-1 | **2.2 (1.2, 4.0)** | **2.2 (1.04, 4.5)** | **4.9 (2.3, 10)** |

**Table F2**. Odds ratios (and 95% confidence intervals) of **MDI 55-69** (when GMFCS < 1) calculated with multinomial logistic regression models as described in Figure 2.

| **Exposure**  **protein** | **hyperEPO only** | **ISSI only** | **hyperEPO+ISSI** |
| --- | --- | --- | --- |
| CRP | 1.3 (0.7, 2.5) | 1.6 (0.9, 3.2) | 1.7 (0.7, 4.2) |
| SAA | 1.0 (0.5, 1.9) | 1.5 (0.7, 3.2) | **3.4 (1.5, 7.6)** |
| MPO | 1.2 (0.7, 2.3) | 0.6 (0.2, 1.4) | 1.2 (0.4, 3.2) |
| IL-1β | 1.1 (0.6, 2.1) | 0.5 (0.2, 1.5) | 1.5 (0.6, 3.6) |
| IL-6 | 1.2 (0.6, 2.3) | 0.8 (0.3, 2.0) | 1.5 (0.6, 3.6) |
| IL-6R | 1.3 (0.7, 2.4) | 0.8 (0.4, 1.6) | 0.9 (0.3, 3.2) |
| TNF-α | 1.1 (0.6, 2.2) | 1.4 (0.7, 3.1) | 2.0 (0.9, 4.3) |
| TNF-R1 | 1.1 (0.6, 2.1) | 0.5 (0.2, 1.4) | 1.5 (0.6, 3.6) |
| TNF-R2 | 1.1 (0.6, 2.2) | 1.8 (0.9, 3.5) | **2.8 (1.2, 6.5)** |
| IL-8 (CXCL8) | 0.9 (0.5, 1.9) | 1.0 (0.4, 2.4) | **2.4 (1.1, 5.2)** |
| MCP-1 (CCL2) | 1.4 (0.7, 2.5) | 1.0 (0.5, 2.2) | 1.2 (0.4, 3.2) |
| MCP-4 (CCL13) | 1.4 (0.8, 2.5) | 0.7 (0.3, 1.5) | 0.8 (0.3, 2.5) |
| MIP-1β *(*CCL4) | 1.3 (0.7, 2.4) | 1.3 (0.7, 2.6) | 2.0 (0.8, 5.3) |
| RANTES (CCL5) | 1.1 (0.6, 2.0) | ***0.4 (0.2, 0.98)*** | 1.2 (0.3, 4.4) |
| I-TAC ( CXCL11) | 1.4 (0.8, 2.6) | 0.7 (0.3, 1.5) | 0.5 (0.1, 2.3) |
| ICAM-1 (CD54) | 1.2 (0.6, 2.3) | 1.1 (0.5, 2.4) | 1.7 (0.7, 4.1) |
| ICAM-3 (CD50) | 1.0 (0.5, 1.9) | ***0.3 (0.3, 0.9)*** | 1.4 (0.6, 3.4) |
| VCAM-1 (CD106) | 1.6 (0.9, 2.9) | 1.2 (0.6, 2.4) | 0.7 (0.2, 2.4) |
| E-SEL (CD62E) | 0.9 (0.5, 1.8) | 1.2 (0.6, 2.5) | **3.0 (1.4, 6.6)** |
| MMP-1 | 1.4 (0.8, 2.6) | 0.8 (0.4, 1.6) | 0.7 (0.2, 2.5) |
| MMP-9 | 1.5 (0.8, 2.6) | 0.7 (0.3, 1.6) | 0.7 (0.2, 2.3) |
| VEGF | 1.3 (0.7, 2.3) | ***0.3 (0.1, 0.8)*** | 0.6 (0.2, 2.1) |
| VEGF-R1 | 1.0 (0.5, 1.9) | 0.7 (0.3, 1.7) | 2.0 (0.9, 4.4) |
| VEGF-R2 | 1.3 (0.7, 2.3) | 0.9 (0.5, 1.9) | 1.5 (0.5, 4.0) |
| IGFBP-1 | 1.3 (0.7, 2.4) | 1.0 (0.4, 2.2) | 1.4 (0.5, 3.9) |

**Table G1. Odds ratios (and 95% confidence intervals) of PDI < 55 calculated with multinomial logistic regression models as described in Figure 2.**

| **Exposure**  **protein** | **hyperEPO only** | **ISSI only** | **hyperEPO+ISSI** |
| --- | --- | --- | --- |
| CRP | **2.3 (1.4, 3.9)** | 1.6 (0.9, 3.1) | **3.7 (1.9, 7.4)** |
| SAA | **2.4 (1.4, 3.9)** | **2.0 (1.04, 3.7)** | **4.3 (2.1, 8.9)** |
| MPO | **2.5 (1.6, 4.1)** | 1.4 (0.7, 2.6) | **2.8 (1.3, 6.0)** |
| IL-1β | **2.5 (1.5, 4.0)** | 1.1 (0.5, 2.3) | **2.4 (1.1, 5.1)** |
| IL-6 | **2.4 (1.4, 4.0)** | 1.8 (0.9, 3.5) | **3.3 (1.7, 6.4)** |
| IL-6R | **2.2 (1.3, 3.5)** | 0.7 (0.3, 1.3) | **2.9 (1.3, 6.3)** |
| TNF-α | **2.0 (1.2, 3.4)** | 1.2 (0.6, 2.4) | **3.7 (2.0, 6.8)** |
| TNF-R1 | **2.5 (1.6, 4.1)** | 0.9 (0.4, 1.8) | 2.0 (0.9, 4.4) |
| TNF-R2 | **2.4 (1.5, 3.9)** | 1.0 (0.5, 2.0) | **2.6 (1.2, 5.4)** |
| IL-8 (CXCL8) | **2.0 (1.2, 3.3)** | 1.0 (0.4, 2.1) | **3.6 (2.0, 6.8)** |
| MCP-1 (CCL2) | **2.7 (1.6, 4.5)** | 1.8 (0.96, 3.4) | **2.8 (1.4, 5.7)** |
| MCP-4 (CCL13) | **2.1 (1.3, 3.5)** | 0.5 (0.3, 1.1) | **2.4 (1.2, 4.8)** |
| MIP-1β *(*CCL4) | **2.4 (1.5, 3.9)** | 0.9 (0.5, 1.8) | **2.5 (1.1, 5.6)** |
| RANTES (CCL5) | **2.2 (1.4, 3.5)** | 0.6 (0.3, 1.2) | **2.5 (1.1, 5.8)** |
| I-TAC ( CXCL11) | **2.4 (1.5, 3.9)** | 0.8 (0.4, 1.5) | 1.9 (0.8, 4.5) |
| ICAM-1 (CD54) | **2.3 (1.4, 3.9)** | 1.8 (0.96, 3.3) | **4.1 (2.1, 8.2)** |
| ICAM-3 (CD50) | **2.3 (1.4, 3.7)** | 0.8 (0.4, 1.8) | **2.6 (1.3, 5.3)** |
| VCAM-1 (CD106) | **2.5 (1.5, 4.0)** | 0.7 (0.4, 1.5) | 1.9 (0.9, 4.0) |
| E-SEL (CD62E) | **2.2 (1.3, 3.6)** | 0.8 (0.4, 1.7) | **3.0 (1.4, 6.1)** |
| MMP-1 | **2.4 (1.5, 3.9)** | 0.7 (0.4, 1.3) | 1.8 (0.8, 4.0) |
| MMP-9 | **2.6 (1.6, 4.2)** | 1.0 (0.5, 2.1) | 1.9 (0.9, 4.4) |
| VEGF | **2.6 (1.6, 4.2)** | 0.7 (0.4, 1.4) | 1.5 (0.7, 3.4) |
| VEGF-R1 | **2.8 (1.7, 4.5)** | 1.0 (0.5, 1.9) | 1.5 (0.6, 4.7) |
| VEGF-R2 | **2.3 (1.4, 3.8)** | 0.9 (0.4, 1.7) | **2.7 (1.2, 5.8)** |
| IGFBP-1 | **2.0 (1.2, 3.3)** | 1.0 (0.5, 2.2) | **4.1 (2.0, 8.2)** |

**Table G2. Odds ratios (and 95% confidence intervals) of PDI 55-69 calculated with multinomial logistic regression models as described in Figure 2.**

| **Exposure**  **protein** | **hyperEPO only** | **ISSI only** | **hyperEPO+ISSI** |
| --- | --- | --- | --- |
| CRP | 1.2 (0.7, 2.1) | 1.6 (0.9, 2.9) | **2.7 (1.4, 5.4)** |
| SAA | 1.1 (0.6, 2.0) | 1.6 (0.9, 3.0) | **3.4 (1.6, 6.9)** |
| MPO | 1.3 (0.8, 2.3) | 1.1 (0.6, 2.0) | 2.0 (0.9, 4.2) |
| IL-1β | 1.1 (0.6, 1.9) | 1.1 (0.5, 2.1) | **2.8 (1.4, 5.4)** |
| IL-6 | 0.8 (0.4, 1.5) | 1.2 (0.6, 2.4) | **3.2 (1.8, 6.1)** |
| IL-6R | 1.2 (0.7, 2.0) | 0.9 (0.5, 1.6) | **2.4 (1.1, 5.3)** |
| TNF-α | 1.1 (0.6, 2.0) | 1.0 (0.5, 2.0) | **2.3 (1.2, 4.4)** |
| TNF-R1 | 1.0 (0.6, 1.8) | 1.0 (0.5, 1.9) | **2.7 (1.4, 5.3)** |
| TNF-R2 | 1.2 (0.7, 2.0) | 0.8 (0.4, 1.5) | **2.2 (1.1, 4.5)** |
| IL-8 (CXCL8) | 1.0 (0.6, 1.9) | 1.0 (0.5, 2.1) | **2.5 (1.3, 4.8)** |
| MCP-1 (CCL2) | 1.2 (0.7, 2.2) | 1.3 (0.7, 2.4) | **2.3 (1.1, 4.6)** |
| MCP-4 (CCL13) | 1.5 (0.9, 2.4) | 0.7 (0.4, 1.3) | 1.1 (0.5, 2.6) |
| MIP-1β *(*CCL4) | 0.9 (0.5, 1.7) | 0.7 (0.4, 1.4) | **3.0 (1.5, 6.1)** |
| RANTES (CCL5) | 1.5 (0.9, 2.4) | 0.6 (0.3, 1.2) | 0.7 (0.2, 2.5) |
| I-TAC ( CXCL11) | 1.4 (0.9, 2.3) | 0.7 (0.3, 1.3) | 1.2 (0.5, 3.0) |
| ICAM-1 (CD54) | 1.0 (0.6, 1.8) | 1.1 (0.6, 2.1) | **3.1 (1.6, 6.1)** |
| ICAM-3 (CD50) | 1.1 (0.6, 1.9) | 0.7 (0.4, 1.3) | **2.1 (1.05, 4.2)** |
| VCAM-1 (CD106) | 1.4 (0.8, 2.3) | 1.2 (0.7, 2.2) | 1.9 (0.9, 4.0) |
| E-SEL (CD62E) | 1.1 (0.6, 1.9) | 1.0 (0.6, 1.9) | **2.7 (1.4, 5.4)** |
| MMP-1 | 1.4 (0.8, 2.3) | 0.8 (0.4, 1.5) | 1.4 (0.6, 3.2) |
| MMP-9 | 1.3 (0.8, 2.2) | 0.8 (0.4, 1.6) | 1.8 (0.8, 3.8) |
| VEGF | **2.6 (1.6, 4.2)** | 0.7 (0.4, 1.4) | 1.5 (0.7, 3.4) |
| VEGF-R1 | 1.5 (0.9, 2.5) | 0.9 (0.5, 1.7) | 1.3 (0.7, 2.8) |
| VEGF-R2 | 1.3 (0.7, 2.2) | 0.8 (0.4, 1.5) | 1.8 (0.9, 3.6) |
| IGFBP-1 | 1.1 (0.7, 2.0) | 1.2 (0.7, 2.1) | **2.9 (1.4, 5.9)** |

**Table H.** Odds ratios (and 95% confidence intervals) of **24 month head circumference Z-score < -2** (when those with a birth head circumference Z-score < -2 are excluded) calculated with logistic regression models as described in Figure 1.

| **Exposure**  **protein** | **hyperEPO only** | **ISSI only** | **hyperEPO+ISSI** |
| --- | --- | --- | --- |
| CRP | 1.8 (0.9, 3.7) | **2.4 (1.1, 4.9)** | **2.8 (1.1, 6.9)** |
| SAA | 1.5 (0.8, 3.1) | 1.7 (0.8, 3.8) | **3.0 (1.3, 7.2)** |
| MPO | 1.5 (0.7, 2.9) | 1.2 (0.5, 2.7) | **2.8 (1.2, 6.6)** |
| IL-1β | 1.8 (0.9, 3.5) | 1.6 (0.7, 3.7) | 2.0 (0.8, 5.1) |
| IL-6 | 1.9 (0.9, 3.9) | **2.8 (1.3, 6.0)** | **2.4 (1.01, 5.5)** |
| IL-6R | **2.0 (1.1, 3.9)** | 1.4 (0.7, 3.0) | 1.2 (0.3, 4.1) |
| TNF-α | 1.2 (0.6, 2.7) | 1.7 (0.7, 3.9) | **3.3 (1.5, 7.1)** |
| TNF-R1 | **2.0 (1.01, 3.9)** | **2.1 (1.03, 4.5)** | 1.8 (0.6, 4.9) |
| TNF-R2 | 1.5 (0.7, 3.2) | **2.3 (1.1, 4.7)** | **3.6 (1.6, 8.3)** |
| IL-8 (CXCL8) | 1.0 (0.4, 2.3) | **2.5 (1.1, 5.6)** | **4.4 (2.1, 9.1)** |
| MCP-1 (CCL2) | 1.9 (0.9, 3.9) | **3.0 (1.4, 6.1)** | **2.6 (1.1, 6.4)** |
| MCP-4 (CCL13) | 1.8 (0.9, 3.4) | 0.8 (0.4, 2.0) | 1.5 (0.5, 4.1) |
| MIP-1β *(*CCL4) | 1.7 (0.9, 3.2) | 0.4 (0.2, 1.3) | 1.2 (0.4, 3.5) |
| RANTES (CCL5) | 1.5 (0.8, 2.9) | 0.6 (0.3, 1.5) | 1.8 (0.6, 5.6) |
| I-TAC ( CXCL11) | 1.6 (0.9, 3.1) | 0.7 (0.3, 1.8) | 1.7 (0.5, 5.1) |
| ICAM-1 (CD54) | 1.8 (0.8, 3.7) | **3.4 (1.7, 6.8)** | **4.0 (1.7, 9.2)** |
| ICAM-3 (CD50) | 1.6 (0.3, 3.3) | 1.1 (0.5, 2.4) | 1.8 (0.7, 4.6) |
| VCAM-1 (CD106) | **2.2 (1.1, 4.3)** | 1.9 (0.9, 3.9) | 1.3 (0.4, 4.0) |
| E-SEL (CD62E) | 1.4 (0.7, 2.9) | 1.5 (0.7, 3.3) | **3.1 (1.3, 7.5)** |
| MMP-1 | 1.6 (0.8, 3.0) | 0.8 (0.4, 1.8) | 1.7 (0.6, 4.6) |
| MMP-9 | 1.3 (0.7, 2.6) | 0.2 (0.1, 1.2) | 2.2 (0.9, 5.5) |
| VEGF | 1.5 (0.8, 2.9) | 0.5 (0.2, 1.3) | 1.7 (0.7, 4.3) |
| VEGF-R1 | **2.1 (1.1, 3.9)** | 1.3 (0.6, 2.9) | 0.7 (0.2, 3.0) |
| VEGF-R2 | 1.7 (0.9, 3.4) | 1.1 (0.5, 2.4) | 1.8 (0.6, 5.0) |
| IGFBP-1 | **2.1 (1.04, 4.1)** | **3.5 (1.7, 7.5)** | 2.2 (0.8, 6.2) |


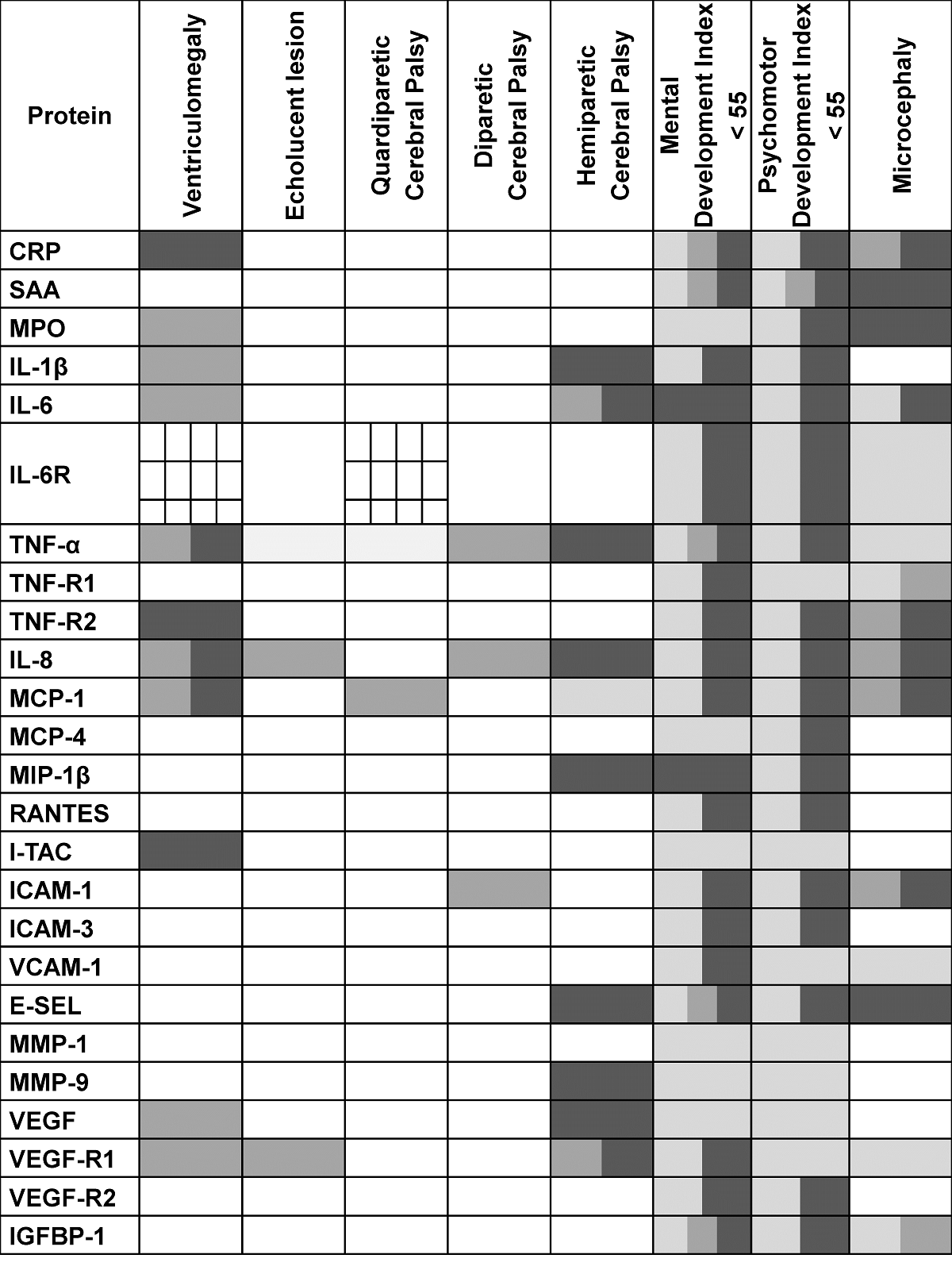


**Figure A. Alternative Figure 5** **Summary of Associations**. We identify three patterns of increased risk of indicators of brain damage associated with hyperEPO and ISSI. hyperEPO only is identified with **light gray**, ISSI only is identified with **middle gray,** and the combination of ISSI+hyperEPO is identified with **dark gray.** Reduced risk of an echolucent lesion associated with hyperEPO only is identified with **cross-hatching (see IL-6R).** In boxes with 2-3 separate shadings, 2-3 patterns were identified. Note: Cells in this table identify patterns of results of unique multivariable regression models fitted to answer whether or not children in each of three mutually exclusive study groups (hyperEPO only, ISSI only, or hyperEPO+ISSI) were at higher or lower risk of the brain damage indicator identified at the top of each column, relative to those in a referent group who did not have hyperEPO or ISSI, adjusting for gestational age category.
